# Student participation in undergraduate medical education: a continuous collective endeavour

**DOI:** 10.1007/s40037-019-00557-0

**Published:** 2019-12-13

**Authors:** Stephanie N. E. Meeuwissen, Jill R. D. Whittingham

**Affiliations:** Department of Educational Development and Research, School of Health Professions Education, Faculty of Health, Medicine and Life Sciences, Maastricht University, Maastricht, The Netherlands

Undergraduate curricular change requires concerted efforts of committed change agents amongst the stakeholders [1]. Publications from the last decade have accentuated that students are important stakeholders in educational design and curriculum change [2, 3]. Striving to a form of student participation demands successful and intense collaboration between staff and students. However, staff-student collaboration has not been found to be self-evident [4]. Individual staff members can form a barrier since collaboration in educational dialogue can be felt to be counterintuitive; it contradicts prevailing conceptions that students do not have the understanding or competencies to inform teaching practice and that staff colleagues and faculty developers are best positioned to do so [4–6]. To raise the bar for student participation, our practice should move beyond traditions of students providing feedback, and move beyond formal inclusion of students in highly structured committees. Although clear structures are desirable for optimal student participation, its institutionalization needs firm cultivation of students’ roles [4]. This requires the development of new traditions in which students are provided the opportunity to be actively involved.

The article by Geraghty et al. ‘Empowering medical students as agents of curricular change: A value-added approach to student engagement in medical education’ [7] presents a highly structured approach to how students can contribute to medical education. The authors describe the implementation of a Student Curricular Board which offers formally trained students opportunities to be involved in evaluation and design of courses and clerkships on the one hand, and in curriculum-overarching committees and large-scale curriculum reforms on the other hand. Geraghty et al.’s paper demonstrates the effort of higher education institutions to respond to the call of student participation, while simultaneously pointing at a need to better facilitate institutes to integrate students into ongoing curricular processes. Some examples are given to move beyond giving feedback and formalities such as involvement in longitudinal curriculum design, pilots for entering residency training, and issues on diversity and wellness [7].

To gain deeper understanding of student participation processes and outcomes, disentanglement of both terminology and approaches is needed [8]. In the literature, a variety of terms are used when discussing student participation, such as ‘student leaders’ and ‘students as agents’ [7, 8]. In this commentary, we use the term ‘active student participation’ to describe a general understanding of student participation that is referred to in many recommendations. The concept of active student participation is described by Bovill and Bulley [9] as follows: *‘Active student participation implies that students are engaged in an experience—whether that is university life, committee representation, or taking part in learning activities’. *Active student participation thus encompasses roles for students such as ‘agents’ or ‘leaders’. This differs strikingly from students’ ‘passive’ role in which students are minimally engaged, turn up for class and absorb knowledge transmitted by teachers [9]. Examples of active student participation in education are characterized by critical questioning, dialogues and discussions that take place in curriculum design and planning, implementation, evaluation and governance [9]. Raising the bar from passive to active student participation has been found to be easier said than done.

The incentives for active student participation are high: students, individual academics, schools and their programs are known to benefit [5, 10], accreditation panels increasingly take into account the extent to which students participate in their schools [2] and the medical education field recognizes schools for excellence in student engagement [2, 11]. Key benefits of student participation and successful student-staff collaboration include enhanced student satisfaction, meta-cognitive understanding of learning and teaching processes, enhanced student–staff relationships and development of personal, professional and academic competences for students [5, 6, 10, 12, 13]. Overall, the benefits contribute to a general development of a (quality) culture within higher education institutions in which student and staff commitment to and motivation for education is enhanced [6, 10, 12, 13]. Notably, educational programs which take active student participation into account have been found to be more often characterized by continuous educational improvement, compared with programs which do not [12]. The other way around also holds: when an institution is committed to continuous improvement, an institutional culture may arise that strives to engage students to improve institutional effectiveness and increase student learning and development [10].

Ultimately, we should strive towards an ongoing collective endeavour, where students and staff work together for excellent education and meaningful preparation of students for careers in the health professions. This requires talented and critical students to be engaged, staff to be empowered and willing to collaborate with students, and structural and cultural embedment of student participation [2, 4, 5, 14]. A collective effort, however, is not yet clearly understood and structural efforts, envisioned and cultivated by the institute and written in policies, are often lacking [5]. We encourage research into optimization of student participation and student-staff collaboration in health professions education. Therefore, more research on student participation is recommended: how can we create a collective endeavour in which all—including students—are continually thinking, working, and learning together?
